# Evolution of mirror-image pain in temporomandibular joint osteoarthritis mouse model

**DOI:** 10.1590/1678-7757-2020-0575

**Published:** 2021-01-25

**Authors:** Nattapon ROTPENPIAN, Sompol TAPECHUM, Anchalee VATTARAKORN, Wongsathit CHINDASRI, Chit CARE, Narawut PAKAPROT, Aree WANASUNTRONWONG

**Affiliations:** 1 Mahidol University Faculty of Medicine Siriraj Hospital Bangkok Thailand Mahidol University , Faculty of Medicine , Siriraj Hospital , Department of Physiology, Bangkok , Thailand .; 2 Prince of Songkla University Faculty of Dentistry Department of Oral Biology and Occlusion Songkhla Thailand Prince of Songkla University , Faculty of Dentistry , Department of Oral Biology and Occlusion , Songkhla , Thailand .; 3 Mahidol University Faculty of Dentistry Department of Oral biology Bangkok Thailand Mahidol University , Faculty of Dentistry , Department of Oral biology , Bangkok , Thailand .

**Keywords:** Mirror-image pain, Osteoarthritis, Complete Freund adjuvant, Temporomandibular joint, Nocifensive behaviors

## Abstract

Mirror-image pain is a kind of pain that occurs on the contralateral side, but its pathogenesis remains unclear.

**Objective:**

To develop an osteoarthritis mouse model for investigating mirror-image pain through observing nocifensive behaviors, histological changes, and nociceptive activity at days 3, 7, 14, 21, and 28 after the chemical induction of unilateral temporomandibular joint (TMJ) osteoarthritis.

**Methodology:**

We randomly divided 6-week-old mice into sham and complete Freund adjuvant groups. To induce nocifensive behaviors, we applied 0.04 g of von Frey filament, 10 psi of air puff, and cold acetone on both sides of whisker pads at different days. The histology of TMJ on both sides was observed by hematoxylin/eosin staining and microcomputed tomography scanning. Furthermore, the nociceptive activity was evaluated using the phosphorylated cyclic AMP response element binding protein (pCREB) and a microglia marker at different days in the trigeminal subnucleus caudalis.

**Results:**

Nocifensive behaviors against mechanical and temperature stimuli on the contralateral side became stronger than the baseline on day 28, in agreement with the elevation of the pCREB and the microglia marker in the trigeminal subnucleus caudalis. Thus, hypernociception on the contralateral side occurred at day 28.

**Conclusions:**

Clearly, the TMJ model with unilateral osteoarthritis exhibited mirror-image pain. Therefore, this model is useful in investigating the pathogenesis of pain and in developing treatments.

## Introduction

Mirror-image pain is the pain found on the opposite side after acquiring peripheral nerve lesion. ^1 , 2^ This type of pain is generally characterized by hypersensitivity to mechanical or thermal painful stimulus that responds even to a light touch or low-threshold stimulus. ^3^ Mirror-image pain occurs in 8%–15% of patients with chronic temporomandibular joint (TMJ) osteoarthritis. ^4^ However, the pathogenesis of mirror-image pain on TMJ osteoarthritis is insufficiently understood. Recent evidence of pain associated with mirror-image pain was mainly found on neuropathic animal models. ^5 , 6^ In chronic constriction injury of the spinal nerve or the infraorbital nerve, pain hypersensitivity on the contralateral side appeared 3 weeks after the nerve lesion. ^7 , 8^ The expression of proinflammatory cytokines (TGFβ1, IL-1β, TNFα, and IL-10) in the contralateral nerve, which was not directly injured, significantly increased 2 weeks after a nerve injury. ^9^ For the pathophysiology of the pain pathway, the contralateral dorsal root ganglia or trigeminal ganglia upregulate the proinflammatory cytokines 3 weeks after the nerve injury. ^8 , 10^ Furthermore, the number of glia cells in the contralateral dorsal horn or trigeminal subnucleus caudalis increases 3 weeks after nerve damage. ^10 , 11^ All of the evidence about mirror-image pain condition in neuropathic pain models suggested that proinflammatory cytokines in the peripheral nerve injury were carried by the cerebrospinal fluid to the contralateral dorsal horn, consequently activating glia cells in that horn. ^12 , 13^ The activated glia cells might potentially stimulate other glia cells and increase the excitability of pain signals in the contralateral dorsal horn. ^5 , 14^

Meanwhile, the association between the structural changes of contralateral nerves, expression of proinflammatory cytokines, and nociceptive activities in the pain mechanisms remains unclear. ^7^ Therefore, an animal model induced by osteoarthritis pain has been proposed to investigate the changes of contralateral structures and the pathophysiology of osteoarthritis-induced mirror-image pain condition.

Several studies on osteoarthritis-induced mirror-image pain condition in animal models obtained consistent results. In a knee joint with osteoarthritis induced by a unilateral complete Freund adjuvant, a proinflammatory cytokine (IL-1β) was highly expressed in the contralateral synovial joint after 3 weeks of induction, but the contralateral knee joint did not morphologically change. ^15 , 16^ Consistent with the previous report, a TMJ model with osteoarthritis induced by a complete Freund adjuvant had high levels of proinflammatory cytokines (IL-6 and TNFα) in the contralateral TMJ, where the bone and cartilage of that joint had remained unchanged on day 27 after the induction by complete Freund adjuvant. ^17^ For the nociceptive activities of pain mechanisms, the marker for nerve injury by activating transcription factor 3 (ATF3) and the proinflammatory cytokines in the contralateral dorsal root ganglia had significantly increased in osteoarthritis knee models after 2 weeks of the induction by complete Freund adjuvant, ^15^ and glia cells had also occurred in the contralateral dorsal horn after 3 weeks in the osteoarthritis model. ^18^ Induction of the proinflammatory cytokines by the unilateral injection of complete Freund adjuvant leads to TMJ osteoarthritis, ^19^ possibly generating mirror-image pain.

Currently, contralateral pain responses and nociceptive activities by bilateral complete Freund adjuvants are fairly available. ^20 , 21^ According to some previous studies, no contralateral pain responses were observed within 2 weeks after administering complete Freund adjuvants in mice. ^22^ Consequently, contralateral pain responses and nociceptive activities at the trigeminal subnucleus caudalis of the spinal tract nucleus related to the TMJ for osteoarthritis-induced mirror-image pain condition, are poorly understood. Therefore, we hypothesized that the development of osteoarthritis-induced pain associated with mirror-image pain can occur after a unilateral complete Freund adjuvant is injected to the TMJ to induce persistent pain in a mouse model. Hence, this study aimed to develop osteoarthritis-induced mirror-image pain in a mouse model to be utilized for future study. We investigated the nocifensive behaviors, the structure of the TMJ, and the activities of the trigeminal pain pathway through the effects of the expression of neuronal and glial markers in the trigeminal subnucleus caudalis of the brainstem on days 3, 7, 14, 21, and 28.

## Methodology

### Study groups and experimental design

This study was approved by the Animal Care and Use Committee, Faculty of Medicine, Siriraj Hospital in Mahidol University (SI-ACUP014/2561) and was conducted according to the Animal Research: Reporting *In Vivo* Experiments (ARRIVE) Guidelines Checklist for animal experiment. ^23^ The sample size was estimated to provide 80% power (1-β) with a 95% confidence interval (α=0.05); six animals per group in different days (days 3, 7, 14, 21, and 28) were required. ^24^ In this study, we used 60 adult male mice (age: 6 weeks, initial body weight: 28–32 g) from the Institute of Cancer Research, considering that they were also used in previous studies investigating orofacial pain caused by infraorbital nerve injury ^25^ and TMJ inflammatory pain conditions. ^26^ These mice were divided into two groups, namely, the sham group (n=30) and complete Freund adjuvant group (n=30). They were experimented at several time points ( Table 1 ). Five mice were housed in each cage, which was temperature controlled (22±2°C) under a 12-hour light/dark cycle and humidity of 45%±15%. They were provided with a solid diet *ad libitum* and were allowed to acclimate for 7 days before the experimental procedure. ^23^

**Table 1:** Number of mice assessed at each time point

Number of mice	Day 3	Day 7	Day 14	Day 21	Day 28
Assessment of nocifensive behaviors	30	24	18	12	6
Structure of the temporomandibular joint	6	6	6	6	6
Nociceptive activity in trigeminal subnucleus caudalis	6	6	6	6	6

### Temporomandibular joint osteoarthritis model induction

Each mouse was anesthetized with sodium pentobarbital (60 mg kg ^-1^ ) intraperitoneally. To induce TMJ osteoarthritis associated with mirror-image pain, we dissolved 10 µl of 1 mg/ml concentration of complete Freund adjuvant (F5881; Sigma-Aldrich, St Louis, MO, USA) in normal saline solution (1:1) and injected the new solution into the right TMJ, while the left TMJ was not injected. The anatomical landmark of injection was prescribed in a previous study. ^26^ To identify the TMJ by palpation, we trimmed the local hairs around the TMJ with a pair of scissors. Then, we inserted a 30-gauge needle through the facial skin until the needle tip reached the zygomatic arch. The needle was slowly moved until it passed under the edge of the arch and ultimately entered into the joint space. When the needle was located in the joint space, the same solution (10 µl) was injected slowly over a period of 5 s using a 30-gauge needle fitted to Hamilton syringe. ^26^ We checked the TMJ location with blue ink injection. The injection of complete Freund adjuvant induces the proinflammatory cytokines, ^17^ which are then expected to degrade the condylar head of the TMJ and subsequently generate mirror-image pain. Moreover, the bodyweight of each mouse was monitored in all experiments.

### Assessment of nocifensive behaviors

**Supplementary Figure 1:**
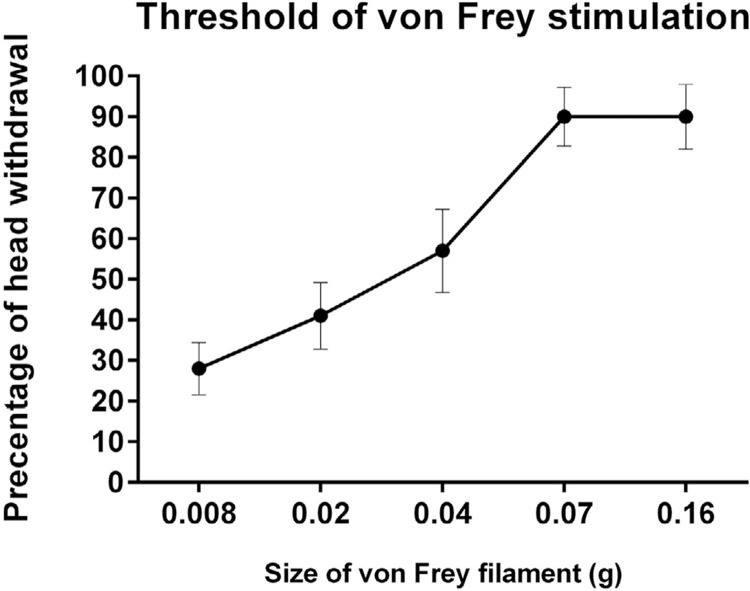
Threshold of von Frey stimulation (n=20 of naïve mice; mean±standard error of mean)

In a previous study, a series of three behavioral tests (0.04 g of von Frey, cold acetone, and 10 psi of air-puff tests) elicited the most remarkable pain-like behaviors for TMJ osteoarthritis and were a dependable and duplicable measurement for pain arising from the injection of complete Freund adjuvant. ^27^ During the assessment of mirror-image pain behaviors, mechanical hyperalgesia, cold allodynia, and mechanical allodynia were tested at the whisker pad on the contralateral side when compared with the ipsilateral sides. Mechanical hyperalgesia was elicited by applying a 0.04 g of von Frey filament on the whisker pads until it was slightly bent. In a pilot study, the optimal von Frey filament size for the threshold of von Frey stimulation was 0.04 g, which is represented in Supplementary Figure 1 . Cold allodynia was induced by applying 0.05 ml of acetone solution on the whisker pads, as modified in a previous study, ^28^ whereas mechanical allodynia was induced by applying 10 psi of air puff, which was controlled by a pneumatic pump, as described in a previous study. ^27^ All responses were recorded within 5 s after application of the stimuli. ^27 , 29 , 30^ Therefore, mice that were alert and awake were individually placed into a plastic restrainer (9 cm×3 cm×3 cm), with their head and front paws exposed outside and their tail firmly fixed with a rubber, to allow them to acclimate in the restrainer for at least 30 min before the test. Nocifensive behaviors were performed on pre-injected mice at day 0 and also on mice on days 3, 7, 14, 21, and 28 after being injected with complete Freund adjuvant. We recorded their responses 12 times by randomly starting and changing either ipsilateral or contralateral side of the whisker pad and taking approximately 10 min per mouse to avoid bias and minimize stress behaviors. ^27^ Subsequently, the responses were scored according to the following criteria: no response=0, head withdrawal=0.25, single face grooming=1, and face grooming more than 3 times=1.5. ^27^ After 12 applications of the three behavioral tests, the pain response scores from each test were summed to achieve the total response pain score. An investigator blinded to the animal group assignment performed all the behavioral tests. Table 1 shows the number of mice at each time point.

**Figure 1:**
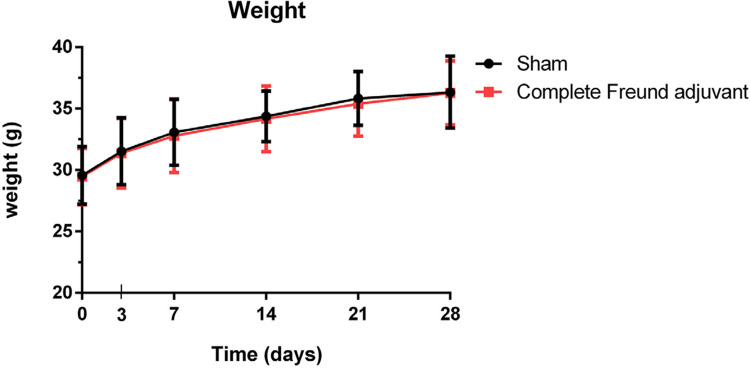
No difference in the bodyweight between the sham and complete Freund adjuvant groups at every time point

### TMJ structure

After nocifensive behaviors, animals were deeply anesthetized and transcardially perfused with 250 ml of ice-cold phosphate-buffered saline with pH 7.4. Then, they were decapitated. The skulls were immersed in 4% paraformaldehyde in 0.1 M of phosphate-buffered saline solution (pH 7.4) for 7 days and then scanned by microcomputed tomography (micro-CT) (30–70 kVp; µCT35 Sanco Medical AG, Bruttisellen, Switzerland) to examine the bone density of the condylar heads of the TMJ. Degeneration of the TMJ and development of osteoarthritis were expected. Condylar heads were imaged from the first part of the condyle to the neck of the ramus. Approximately 150–250 images were obtained from each mouse. ^31^ Six mice in each group were dividedamong each time point (days 3, 7, 14, 21, and 28).

After micro-CT scanning, we removed the skin on the head and opened the cervical bone. Thereafter, we dissected the skull that covers the cerebrum and removed the brain and the spinal cord. In the dissected brain and spinal cord, only the trigeminal subnucleus caudalis was removed. Next, the TMJ was dissected into two separate sides, namely, ipsilateral side and contralateral side. The TMJs were then dissected and decalcified in 10% formic acid for 7 days before being embedded in paraffin. The TMJ structures including the condylar head, articular disc, and temporal bone were sagittally sectioned into 10 µm sections using a microtome and stained with hematoxylin/eosin. ^20^ The sections were collected at intervals of approximately one in every 10 sections to observe the TMJ surface changes. Furthermore, we assessed and graded the degree of joint degeneration individually and independently using the Osteoarthritis Cartilage Histopathology AssessmentSystem ^19 , 32^ observing the following criteria: surface intact/cartilage morphology intact=0, surface intact with abrasion on the superficial layer=1, surface discontinuity=2, vertical fissures or clefts=3, surface erosion=4, sclerotic bone within a denuded surface=5, and deformation=6. ^33^ Each datum was blindly analyzed by the mean of the grade.

### Nociceptive activity in trigeminal subnucleus caudalis

After introducing nocifensive behaviors and investigating TMJ degeneration, we removed trigeminal subnucleus caudalis of both groups on days 3, 7, 14, 21, and 28, and further immersed it in 4% paraformaldehyde with 0.1 M of phosphate-buffered saline (pH 7.4). The trigeminal subnucleus caudalis samples were embedded in the paraffin section and sliced into 4 µm-thick sections by using a microtome. Every 10 ^th^ section was processed to examine the nociceptive activity in the trigeminal subnucleus caudalis, which demonstrated the mirror-image pain. The pCREB and the microglia marker OX42 represented the nociceptive activity by immunofluorescence and immunohistochemistry staining, respectively. We validated the specificity of the primary antibody by using positive and negative controls in our staining; thus, the sections were immunolabeled with rabbit monoclonal pCREB diluted at 1:200 (SC-52; Santa Cruz Biotech, Dallas, TX, USA). For the secondary antibody, we used an Alexa Fluor 488 conjugated goat anti-rabbit IgG diluted by 1:500 (Jackson Immuno Research, West Grove, PA, USA). These sections were measured by fluorescence microscopy. Regardless of the staining intensity, each green dot at the ipsilateral and contralateral sides of the trigeminal subnucleus caudalis was termed as pCREB positive. For OX42 representing microglial expression, the sections were immunolabeled with rabbit polyclonal OX42 diluted at 1:200 (SC-52; Santa Cruz Biotech, Dallas, TX, USA) and then subjected to Dako EnVision and Peroxidase (DC EnVision System, HRP, CA, USA). Subsequently, the sections were examined by light microscopy (Olympus, Tokyo, Japan). Each brown dot at the ipsilateral and contralateral sides of the trigeminal subnucleus caudalis was termed as microglia positive, irrespective of the staining intensity. We then counted pCREB and microglia in nine sections per mouse. Each group has six mice for the different time points. Using the brain atlas of Paxinos and Keith ^34^ (2001), we assessed the histology of the trigeminal subnucleus caudalis.

As mentioned, the nociceptive activity in the trigeminal subnucleus caudalis was evaluated individually and independently, and each datum was blindly analyzed by the means number of pCREB and microglia per section at each time point for the two groups.

### Data analysis

All statistical data were analyzed by the Statistical Package for the Social Sciences (SPSS) version 26.0 (IBM, Chicago, IL, USA). Data are expressed as the mean ± standard error of the mean. The normal distribution of data was examined using the Kolmogorov–Smirnov test. We compared two independent groups in terms of the body weight, nocifensive behaviors, and nociceptive activity by using the independent *t* -test. In comparing TMJ degeneration between more than two independent groups, we used the one-way analysis of variance (ANOVA) and Dunnett’s test (post hoc test) sequentially. Moreover, the correlations of two independent groups in terms of nocifensive behaviors and nociceptive activity were analyzed by Pearson’s correlation. A p value of less than 0.05 indicated statistical significance.

## Results

### Effects of the injection of complete Freund adjuvant on animal bodyweight

As explained, our experiment was performed at several time points. Figure 1 illustrates all data at each time point. The injection of complete Freund adjuvant certainly induced TMJ osteoarthritis associated with mirror-image pain, without affecting mouse’s bodyweight. During the 28 days of evaluation, all mice exhibited normal social behavior, normal food intake, and no stress behaviors.

### Development of mirror-image pain-like behaviors 28 days after the injection of complete Freund adjuvant

**Figure 2:**
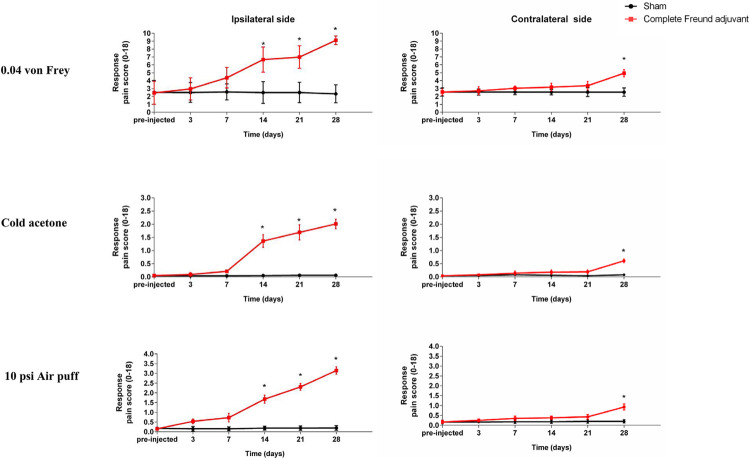
Development of mirror-image pain-like behaviors 28 days after the injection of complete Freund adjuvant. The line graph shows the mean pain response score of three stimuli, namely, 0.04 g of Von Frey, cold acetone, and 10 psi of Air-puff tests, for the two mouse groups. *p<0.05: sham vs. complete Freund adjuvant by independent t-test in each time point; n=30 on pre-injection and day 3, n=24 on day 7, n=18 on day 14, n=12 on day 21, and n=6 on day 28.

Figure 2 shows the development of mirror-image pain-like behaviors using three stimuli. On day 14, all three stimuli showed significant initial pain-like behaviors on the ipsilateral side (n=18, p< 0.05). Considering the injection of complete Freund adjuvant, the pain persisted, leading to the significant development of mirror-image pain-like behaviors 28 days after the injection (n=6, p<0.05).

### Development of osteoarthritis in the temporomandibular joint 21 days after injecting complete Freund adjuvant

**Figure 3:**
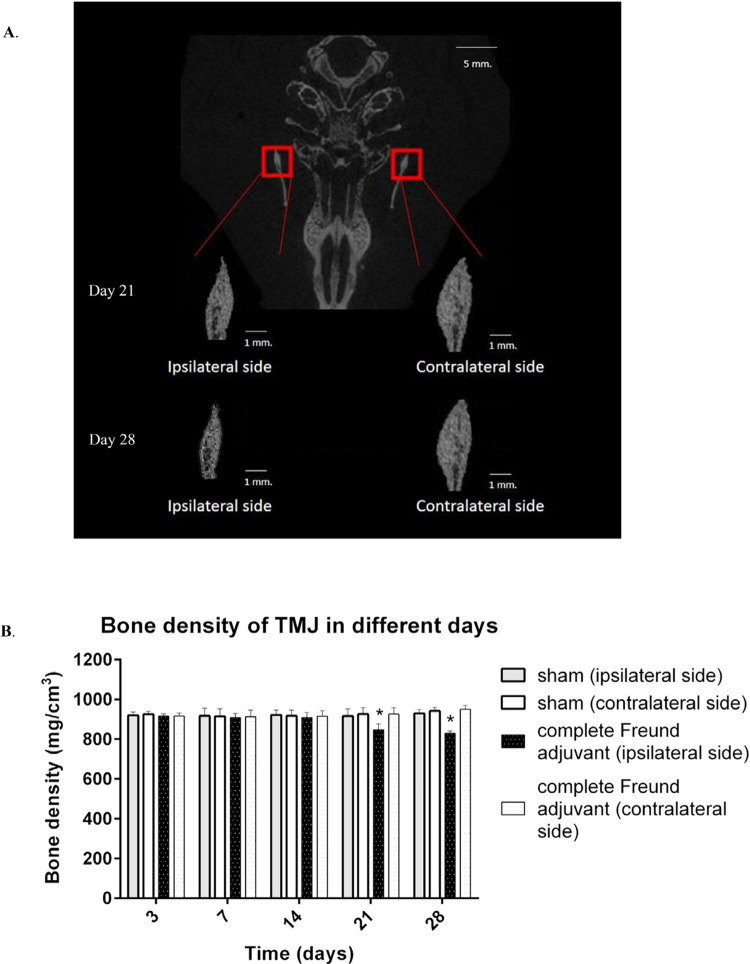
Injection of complete Freund adjuvant causes anatomical changes of the condylar head of temporomandibular joint. (A) A representative image of microcomputed tomography scans of the temporomandibular joint at days 21 and 28, with scale bars of 5 mm of the upper picture and 1 mm of the inset box. (B) The bar graph shows the mean bone density of the ipsilateral and contralateral sides, * p<0.05: sham vs. complete Freund adjuvant by one-way analysis of variance (ANOVA) and Dunnett’s test (post hoc test) sequentially in each time point, n=6

Figure 3 shows the anatomical changes of the condylar head of the TMJ on the ipsilateral side. After 21 and 28 days, the mean bone density of the ipsilateral condylar head in the complete Freund adjuvant group was significantly less than that in the sham group (n=6, p<0.05). For the contralateral condylar head, the bone density remained unchanged at any time point ( Figure 3B ).

**Figure 4:**
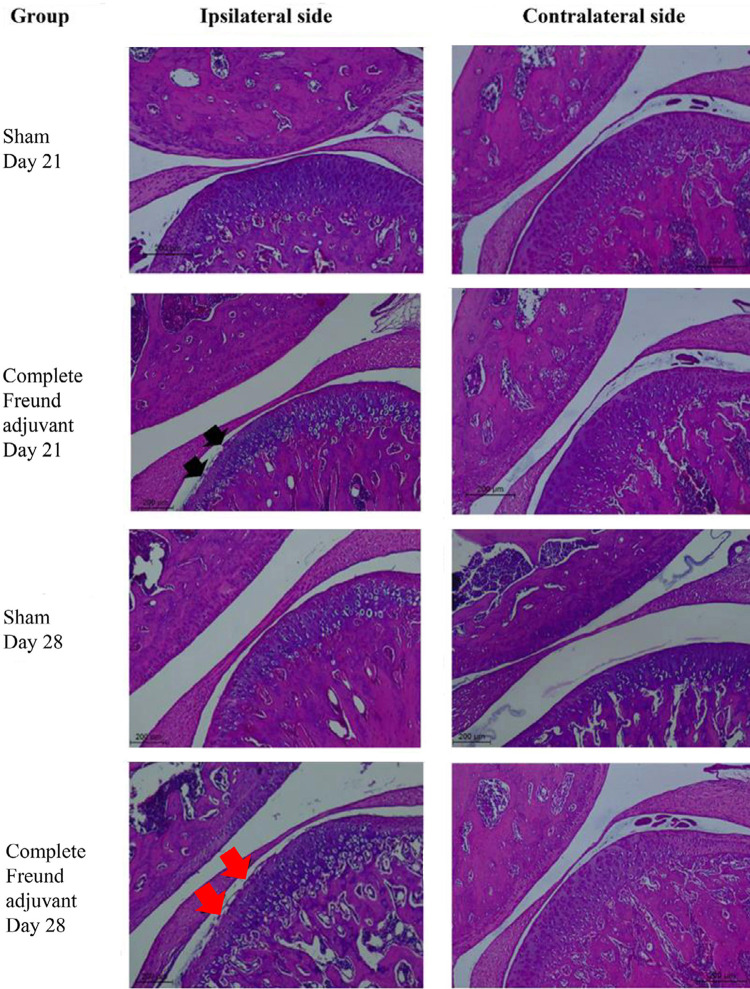
Representative image of hematoxylin/eosin staining of the condylar head of the temporomandibular joint at days 21 and 28, with a scale bar of 200 µm. The black arrow represents grade 3 at day 21 after complete Freund adjuvant injection, whereas the red arrow represents grade 4 at 28 day after the injection. Both sides for the sham group and contralateral side of the complete Freund adjuvant group obtained grade 0, n=6

The micro-CT scan results confirmed that the surface of the ipsilateral condylar head of the TMJ eroded and was detached. Based on the Osteoarthritis Cartilage Histopathology Assessment system, the grade for the ipsilateral side was 3.7±0.33 and 3.8±0.42 after 21 and 28 days from the day the complete Freund adjuvant was injected, respectively (n=6). Meanwhile, the grade on both sides of the sham group and the contralateral side of the complete Freund adjuvant group was grade 0, indicating that the surface of the condylar head of the TMJ remained intact ( Figure 4 ).

### Complete Freund adjuvant induced the nociceptive activity on the trigeminal subnucleus caudalis

**Figure 5:**
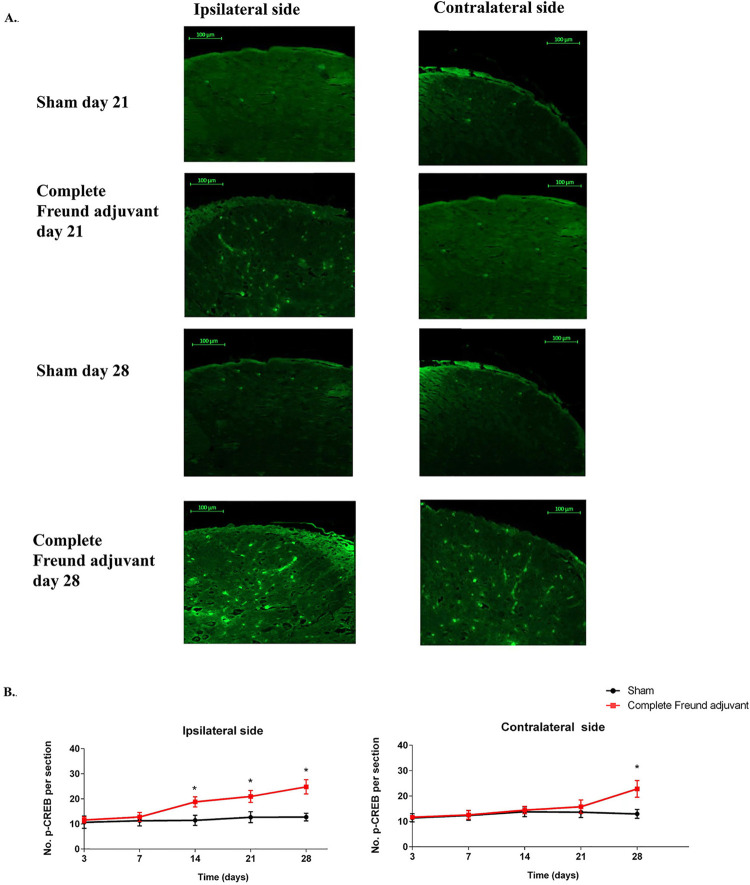
Expression of pCREB induced by complete Freund adjuvant on the ipsilateral and contralateral sides of trigeminal subnucleus caudalis. (A) Representative image of pCREB on the ipsilateral and contralateral sides of trigeminal subnucleus caudalis on days 21 and 28, with a scale bar of 100 µm, representing mirror-image pain on the trigeminal subnucleus caudalis. (B) The line graph shows the mean of pCREB expression per section on the ipsilateral and contralateral sides of trigeminal subnucleus caudalis in the sham and complete Freund adjuvant groups on days 3, 7, 14, 21, and 28, *P<0.05: sham vs. complete Freund adjuvant by independent t-test in each time point, n=6

**Figure 6:**
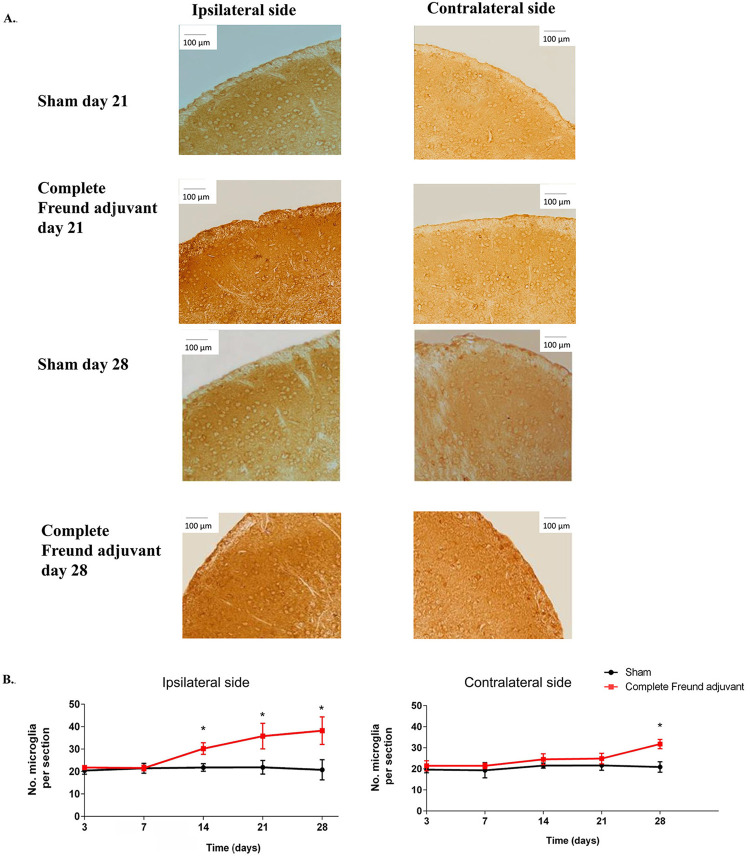
Microglial expression induced by complete Freund adjuvant on the ipsilateral and contralateral sides of trigeminal subnucleus caudalis. (A) Representative image of microglia of the ipsilateral and contralateral trigeminal subnucleus caudalis on day 28, with a scale bar of 100 µm, representing mirror-image pain on the trigeminal subnucleus caudalis. (B) The line graph shows the mean of microglia expression per section on the ipsilateral and contralateral sides of trigeminal subnucleus caudalis in the sham and complete Freund adjuvant groups on days 3, 7, 14, 21, and 28, *P<0.05: sham vs. complete Freund adjuvant by independent t-test in each time point, n=6

The central nociceptive activity was enhanced after the induction of complete Freund adjuvant on the trigeminal subnucleus caudalis, as showed in Figures 5 and 6 . The nociceptive activity on the trigeminal subnucleus caudalis was associated with increased pCREB and microglia expression, which indicated strengthening of the excitatory synapses and development of pain-like behaviors. pCREB- and microglia-positive neurons were observed both in the ipsilateral and contralateral trigeminal subnucleus caudalis ( Figures 5A and 6A ). The mean number of pCREB and microglia on the ipsilateral side in the complete Freund adjuvant group was significantly higher than that in the sham group on days 14, 21, and 28, respectively (n=6, p<0.05) ( Figures 5B and 6B). Likewise, the mean number of pCREB and microglia on the contralateral side was signiﬁcantly higher in the complete Freund adjuvant group than in the sham group on day 28 (n=6, p<0.05) ( Figures 5B and 6B ).

The correlation between contralateral nocifensive and nociceptive activity was estimated as Pearson’s correlation coefficient ( *r* )≥0.9. The contralateral nociceptive activity on the trigeminal subnucleus caudalis induced by the injection of complete Freund adjuvant was consistent with the mirror-image pain-like behaviors 28 days after the injection.

## Discussion

Our study shows the development of mirror-image pain on TMJ osteoarthritis in mice induced by the unilateral injection of complete Freund adjuvant. During the period, the ipsilateral and contralateral nocifensive behaviors of mice were not affected by food and water intake, as confirmed by the lack of difference in bodyweight. After innocuous stimuli, including injection of complete Freund adjuvant, were introduced to the contralateral whisker pads, the mice developed nocifensive behaviors on day 28 despite the absence of osteoarthritis on the contralateral TMJ. The bone density of contralateral TMJ remained unchanged on day 27 after the induction of complete Freund adjuvant. ^17^ This finding agrees with other studies investigating on pain associated with mirror-image pain using a nerve injury model, suggesting that pain-like behaviors start to occur on the ipsilateral side and contralateral side approximately 2–3 weeks and 3 weeks after nerve injury, respectively. ^7^

In addition to nocifensive behavior changes, immunohistochemistry revealed pCREB elevation in the trigeminal subnucleus caudalis, consistent with other mirror-image pain studies. In studies using a nerve injury model, pCREB expression was higher in the ipsilateral spinal cord 2 weeks after the injury than that 4 weeks after the injury in the contralateral spinal cord. ^35^ When postsynaptic receptors, such as glutamate receptors, on the neuronal membrane in the trigeminal subnucleus caudalis are activated, calcium and sodium influx occurs, resulting in the sequential increase of cAMP and pCREB expression. ^35^ Thus, pCREB represents noxious stimulation that reached the trigeminal subnucleus caudalis. It serves as a key step in the development of activity-dependent synaptic plasticity in the spinal cord and trigeminal subnucleus caudalis, ^35^ leading to a higher frequency of action potentials and subsequent enhancement of pain signals to the cerebral cortex. ^36^ Furthermore, pCREB indicates the transcription of various proinflammatory and/or excitatory factors. However, this fact alone does not support the possible mechanism of the osteoarthritis-induced mirror-image pain. Moreover, the complete Freund adjuvant itself did not activate pCREB expression in the trigeminal subnucleus caudalis on both sides. However, persistent TMJ inflammation induced by complete Freund adjuvant is the result of pCREB expression. Thus, the possible mechanism of mirror-image pain is the maintenance of the hypernociceptive state, resulting in sensitization of the central nervous system through microglial cell signaling.

Monoclonal antibody OX42 binds to CD11b, which is a beta-incretin marker found in microglia. This protein is upregulated when microglia are activated. ^37^ After tissue damage, microglia are the first to become activated and remain for 3–4 weeks on both ipsilateral and contralateral sides of the spinal cord and trigeminal subnucleus caudalis. ^38^ Microglial activation contributes to the release of proinflammatory cytokines and chemokines, leading to widespread inflammatory responses. ^2^ Released inflammatory mediators serve as messenger molecules that mediate the communication between cells in the immune system in other body parts, particularly on the same structure on the opposite side. ^39^ Microglia cells in the contralateral dorsal horn are reportedly increased after 3 weeks in an osteoarthritis model. ^38^ Therefore, this previous study assessed OX42 immunoreactivity to indirectly determine microglial activation. ^37 , 38^ In the current study, OX42 immunoreactivity in the trigeminal subnucleus caudalis on both sides was elevated in this model of TMJ osteoarthritis induced by complete Freund adjuvant. The microglia activation in the contralateral trigeminal subnucleus caudalis might cause pain sensitization, resulting in nocifensive behaviors associated with mirror-image pain when innocuous stimuli were introduced.

Taken together, the unilateral injection of complete Freund adjuvant into the TMJ successfully induced osteoarthritis in the ipsilateral TMJ but not in the contralateral TMJ. ^22^ However, pain associated with mirror-image pain could be observed in behavioral and immunohistochemistry tests, and the pathological condition on the ipsilateral side may spread to the opposite side. Other pathogeneses such as descending pain modulation and greater loading in the opposite side when ipsilateral pain persists may be involved in the development of mirror-image pain. Moreover, the GABAergic neurons in the dorsal horn of the spinal cord had a significantly decreased expression and function in pain induced by complete Freund adjuvant at the knee and hip joints. ^36^ In patients with TMJ osteoarthritis pain, the neurons on both sides of the brainstem might decrease, especially at the raphe nucleus, which is a nucleus for descending pain modulation; thus, neuronal reduction in the raphe nucleus might increase pain sensation in these patients. ^40^ Moreover, the functional loading of force occurs in the pathologic side, leading to the overfunction and compensation of the opposite joint. ^40^

Mirror-image pain in TMJ osteoarthritis started at day 28. However, the bony change of ipsilateral condylar TMJ initially developed at day 21. If mirror-image pain can be treated, the treatment should start at day 14, when ipsilateral osteoarthritis starts to develop. However, further evaluation of the mechanisms is needed.

Meanwhile, the limitation of our study includes the subjective definition of pain, and in experimental animals, we can only observe their behaviors as a result of activating the nociceptive pathways. Hence, our study associated patient condition with the behavioral nociception of mice.

In brief, our study provides an insight on the possible development of TMJ osteoarthritis that induced mirror-image pain in a mouse model to achieve contralateral nocifensive behaviors after ipsilateral pain occurrence. These findings are useful in future pathogenesis studies of mirror-image pain models.
